# Plasma-generated reactive water mist for disinfection of N95 respirators laden with MS2 and T4 bacteriophage viruses

**DOI:** 10.1038/s41598-022-23660-5

**Published:** 2022-11-19

**Authors:** Jinjie He, Michael Waring, Alexander Fridman, Alexander Rabinovich, Charles Bailey, Gregory Fridman, Christopher M. Sales

**Affiliations:** 1 Department of Civil, Architectural, and Environmental Engineering, Drexel University, Philadelphia, PA USA; 2 C. & J. Nyheim Plasma Institute, Drexel University, Camden, NJ USA; 3 Department of Mechanical Engineering and Mechanics, Drexel University, Philadelphia, USA; 4 AAPlasma LLC, Philadelphia, PA USA

**Keywords:** Infectious diseases, Viral infection, Occupational health

## Abstract

Due to the shortage of personal protective equipment (PPE) during the COVID-19 pandemic, the interest and demand for sterilization devices to reuse PPE has increased. For reuse of face masks, they must be effectively decontaminated of potential infectious agents without compromising its filtration ability during sterilization. In this study, we utilized an atmospheric pressure pulsed dielectric barrier discharge (DBD), combined with nebulized liquid microdroplets to generate plasma-activated mist (PAM). MS2 and T4 bacteriophages were used to conduct the decontamination tests on two types of N95 respirators. Results showed at least a 2-log reduction of MS2 and T4 on N95 respirators treated in one cycle with 7.8% hydrogen peroxide PAM and at least a 3-log reduction treated in 10% hydrogen peroxide PAM. In addition, it was found that there was no significant degradation in filtration efficiency of N95 respirators (3M 1860 and 1804) treated in 10% hydrogen peroxide PAM found after 20 cycles. In terms of re-useability of masks after treatment as determined, it was shown that the elastic straps of 3M 1804 were fragmented after 20 treatment cycles rendering them unusable, while the straps of 3M 1860 were not negatively affected even after 20 disinfection cycles.

## Introduction

Since the first case was identified in December 2019, coronavirus disease 2019 (COVID-19) has spread rapidly in multiple countries and was declared a pandemic by the World Health Organization in March 2020^1^. The virus that causes the disease COVID-19 is called severe acute respiratory syndrome coronavirus 2 (SARS-CoV-2) and can spread among humans via respiratory droplets from coughing and sneezing, aerosols from breathing and talking, and fomites^2^. Previous research has encouraged face masks (also referred to as respirators) be worn in publics to limit the spread of the COVID-19^3,4^. Surgical mask can block large-particle droplets, splashes, sprays, or splatter that may contain germs (viruses and bacteria), while N95 respirator can efficiently filtrate airborne particles at least 95%. The edges of the N95 respirator are designed to fit wearer’s face and avoid air that contains contaminant to get to the wearer’s nose and mouth without filtration through the respirator^5^. In addition to face masks, other personal protection equipment (PPE) such as gloves, face shields and gowns were also in high demand throughout parts of the globe as COVID-19 cases continue to increase^6^. Early in the pandemic, the supply and distribution of PPEs have not kept up with their high demand, forcing healthcare workers and first responders to reuse PPEs, such as surgical and N95 respirators.

Due to the shortage of PPE, especially N95 respirators, at the beginning of the COVID-19 pandemic, a wide range of studies focusing on decontamination and reuse of PPE were conducted, including the use of ultraviolet germicidal irradiation (UVGI), vaporized hydrogen peroxide (VHP), ethylene oxide (EtO), microwave oven, bleach, heat treatment, ethanol, liquid hydrogen peroxide, autoclave, isopropyl alcohol, wipe products, tap water, soap and water, and traditional electric rice cooker^7^. It is not straightforward to compare the efficacy of the different approaches because different viruses and bacteria were tested by each method as well as on various mask materials. For example, UVGI can reduce at least 3-log of H1N1 influenza^8^, while VHP can achieve 6-log of *Geobacillus stearothermophilus* spores on N95 respirator^9^. However, some of the methods, such as UVGI, VHP, microwave oven, bleach, heat treatment and autoclave, decrease the quality of N95 respirator by lowering the filtration efficiency or compromising the material integrity of the mask straps^7^.

Non-thermal plasma, including direct exposure and remote exposure, as a low temperature method for sterilization of surfaces and materials has been shown to be efficient for microbial decontamination. Studies have shown that plasma technologies can inactivate pathogens on the surface of medical devices^10^ and agricultural products^11^. In relation to the COVID-19 pandemic, recent research has demonstrated that direct non-thermal plasma can inactivate SARS-CoV-2 RNA in bioaerosols^12^. In another study, they were able to demonstrate the use of surface dielectric barrier discharge (DBD) to inactivate a pseudovirus with the SARS-CoV-2 S protein in cold-chain storage and transportation environments^13^. In addition to applying plasma directly to materials for disinfection, it has also been widely shown that plasma-activated water (PAW) can act as an effective bactericidal solution^14,15^. A more gentle and versatile approach akin to PAW is the production of PAM where nebulized droplets of water or other solutions are exposed to plasma discharge. Rather than submerging materials in PAW, PAM methods produce droplets that can carry plasma-generated reactive chemical species with microbial and viral inactivation ability to sterilize surfaces. A prior study had shown that PAM accumulates high concentrations of hydrogen peroxide and acquires an acidic pH, which creates suitable conditions within PAM for potent antimicrobial activity^16^. Reactive oxygen species (ROS) and reactive nitrogen species (RNS) are considered the most important role of disinfection in PAM^17^. ROS mainly includes radicals, hydrogen peroxide, singlet oxygen, superoxide anions, and ozone, whereas RNS mainly includes nitrate, nitrite, peroxynitrite, nitric oxide radical, ammonia, and nitrogen^15^. These ROS and RNS have been shown to react with virus DNA (double and single strand) and RNA^18,19^. For example, a previous study showed that ROS- and RNS-containing PAM can inactivate bacteria on the surface of kale without damage of surface itself^20^. Therefore, PAM may have the potential to inactivate SARS-Cov-2 on the surface of N95 respirators without decreasing its ability to protect the user. Moreover, the combination of 7.8% aerosolized hydrogen peroxide and plasma enhanced the efficacy of the inactivation of *Salmonella* and *L. innocua* on the surface of fresh produce^21^. This combination may also be an effective method to treat PPE.

In this study, we aimed to test the microbial decontamination efficiency of MS2 and T4, both non-enveloped coliphages, on two types of N95 respirators (3M 1804 and 1860) by applying PAM, when the solution being nebulized to create PAM were either deionized (DI) water or water containing varying concentrations of H_2_O_2_. We also wanted to test the filtration efficiency using particle size analysis to see if there is any negative effects on the material performance of the N95 masks.

## Materials and methods

### Plasma system

**Figure 1:**
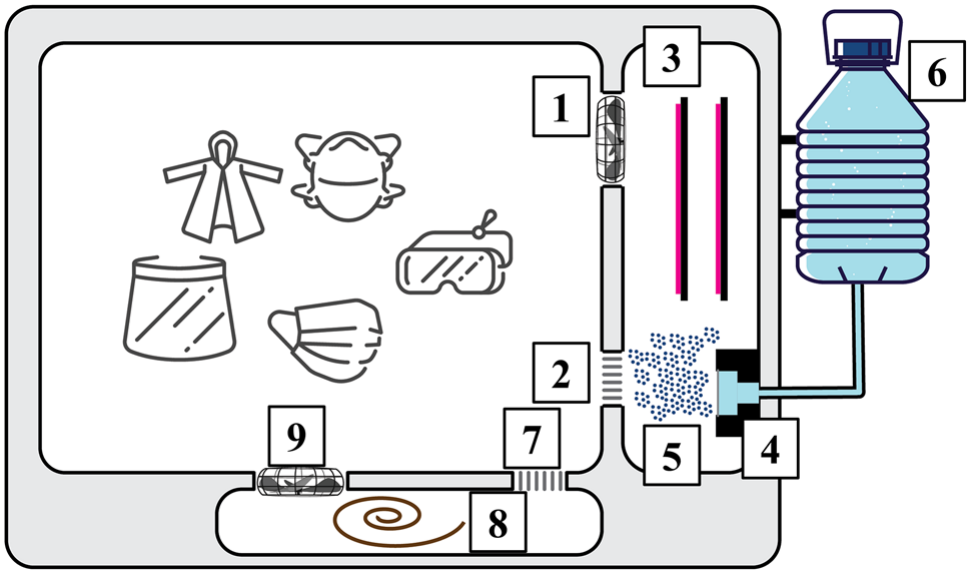
Schematic of plasma system. It consists of three chambers: large 53-L open area for the PPE, the plasma chamber (#1–6) and heating chamber (#7–9): 1. Fan forcing the air into the plasma chamber. 2. Exit from the plasma chamber where droplets, mixed with plasma-generated reactive oxygen and nitrogen species (RONS) are introduced into the area with the PPE. 3. Plasma is generated with a 20 kHz, 3,000 V dielectric barrier discharge. 4. Ultrasonic nebulizer is used to generate water microdroplets with mean diameter of ~ 5 µm. 5. Water microdroplets form a dense fog that is produced immediately after the plasma zone and mixed with RONS. 6. Each cycle consumes 1 ml of DI water or other solutions and our current prototype has a 750 ml container. 7. For the heated air cycle, the air (containing residual RONS and moisture) is sucked in from the chamber with the PPE. 8. The air-RONS-moisture is heated to 50 °C to facilitate removal of the residual reactive chemistry and drying of the PPE. 9. Warm air is forced into the chamber with the PPE by a fan.

The prototype was made from a 53 L electric dish dryer (Fig. 1). A 20 kHz 3,000 V surface DBD was installed outside the dish dryer and connected to the chamber inside. A 750 mL water container was fixed on the side of dish dryer and was connected to the ultrasonic nebulizer that was linked to the DBD reactor.

A pre-set program was installed in the system through Arduino. The program was used to control operation time of DBD reactor, fan, nebulizer, and the drying system. In this study, we set the disinfection process in 3 stages for 20 min total. The first stage was set as 5 min DBD reactor, fan and nebulizer on; the second stage was 5 min DBD reactor and fan operating without the nebulizer; and only the drying system is on during the third stage for 10 min. During the drying cycle, the temperature at the exit of the heating zone was increased to 50 °C by flowing air using a standard computer fan at approximately 30 CFM over a resistive heating coil. Voltage to the coil was monitored by an Arduino microcontroller and raised or lowered to maintain the chamber temperature at the preset 50 °C.

### Bacteriophage

The working solution of two bacteriophage viruses, MS2 (ATCC 15597-B1) and T4 (ATCC 11303-B4) were stored at 4 °C before testing. The concentration of stock was quantified by plating appropriate dilutions by the overlay plaque assay method on LB Agar^22^. Briefly, underlay agar plates were made from LB agar (MP Biomedicals). The top agar was made from the same media type but with lower concentration of agar. The mixture of 100 μL MS2 or T4 stock, 100 μL the host (*Escherichia coli* MG1655) and 5 mL of molten top agar were plated on the top of underlay agar. After the top agar solidified, plates were incubated at 37 °C overnight.

### Decontamination test

Two commonly used N95 respirators (3M 1804 and 3M 1860) were tested in this study. Two sides of 3M 1860 were tested because they were made of different materials. N95 respirators were cut into 1 × 6 cm coupons. 100 μL of virus stock were spot-inoculated onto each coupon. Coupons were left in a laminar flow safety cabinet until dry (approximately 3 h) before treatment.

Following, the inoculated coupons were treated by our system and analyzed for microbial inactivation. Three to six replicates of coupons were treated for each condition. Hydrogen peroxide (7.8% and 10%) or de-ionized (DI) water were used to generate mist. Different concentration of hydrogen peroxide was diluted from 35% hydrogen peroxide (Arkema). Negative control was also conducted in each experiment.

### Phage recovery from coupons and quantification

After treatment, T4 or MS2 was recovered from face mask materials by shaking or vortexing in 10 or 15 mL of sterile phosphate buffered saline (PBS). N95 1860 outer layer and 1804 coupon were extracted by 15 mL of PBS; N95 1860 inner layer was extracted by 10 mL of PBS. Shaking for 20 min at 200 rpm performed a higher recovery efficiency than vortex for 1 min, so shaking was used in most of the experiments in this study (supplementary material Recovery efficiency of MS2 and T4 from N95 respirator material).

Double agar overlay plaque assay was used to quantify viable viruses recovered in terms of plaque forming units (PFUs). No treatment controls were also performed to account for any loss in viable viruses (PFUs) not associated with treatment in our system.

Decontamination efficiency was determined using the following equation, where N_treated,avg_ is the average PFU/coupon of the treated coupons, and N_untreated,avg_ is the average PFU/coupon of the untreated coupons.

1 $${\text{Decontamination efficiency }} = {\text{ log }}\left( {{\text{N}}_{{{\text{untreated}},{\text{avg}}}} /{\text{ N}}_{{{\text{treated}},{\text{avg}}}} } \right)$$

### Chemical properties of plasma-activated mist and conditions in the chamber

The chemical properties of PAM were determined by measuring pH (Hydrion, range 0–3 and range 0–6), NO_3_^−^ (Quantofix, range 10–500 mg/L), NO_2_^−^ (Quantofix, range 1–80 mg/L) and total peroxide (Quantofix, range 50–1000 ppm) via commercial test strips. Test strips were placed in the middle of chamber of our system during one decontamination cycle. After the cycle finished, test strips were read by comparing the color table provided by the manufacturers. Condensed water was collected by covering aluminum foil on the exit of plasma chamber for 1 cycle treatment (#2 in Fig. 1) and then measured pH, NO_3_^−^ and NO_2_^−^ via test strips.

Temperature and humidity were measured by a data logger (Elitech GSP-6G). The probe was kept in the middle of the chamber while operating. Ozone was measured using 254 nm narrow wavelength UV absorption (model 106-M ozone monitor, 2B Technologies, Boulder).

### Mask filtration efficiency test

The filtration efficiency of N95 respirators was conducted before and after different numbers of cycles treatment (1 or 20 cycles) in our system. Particles were generated in a 400 L stainless steel chamber, and by counting particle concentrations in an alternating manner between air sampled (*i*) directly from the chamber and (*ii*) through the filter media. The removal efficiency of N95 respirators was computed by using Eq. (1):

2 $$\eta = 1 - \frac{{C_{{{\text{filter}}}} }}{{C_{{{\text{chamber}}}} }}$$

where *η* is the filter removal efficiency; and *C*_chamber_ and *C*_filter_ (#/cm^3^) are the particle concentrations in the air from the chamber and through the filter media, respectively.

Particles less than ~ 1 μm were generated using aerosolized sodium chloride (NaCl) via an aerosol generation system consisting of these items in series: an atomizer with a solution of DI water and sodium chloride (NaCl) to generate NaCl particles (TSI Aerosol Generator 3076); a diffusion dryer to remove water from the airstream (TSI Diffusion Dryer 3062); and a neutralizer to apply a standard, equilibrium charge distribution (TSI Aerosol Neutralizer 3077A). Chamber air was mixed with fans so that concentrations were uniform. Tested filter media were held in a 3D-printed filter housing. Flow rates were designed such that the face velocity through the filter media was 10 cm/s. Particles were counted with a TSI Fast Mobility Particle Sizer (FMPS) 3091, which measures size distributions each second using 32-channels between the diameter size range of 0.0056 and 0.56 μm. The outcome of this procedure, for each tested filter media, is a determination of the filter removal efficiency for total particles and as a function particle size (with diameters between 0.0056 and 0.56 μm). Results from this procedure allow comparison with those of an N95 test, which tests for 95% removal efficiency of 0.3 μm diameter particles.

### Statistical analysis

Data sets were analyzed using SPSS. The P-value of < 0.05 is considered significant. The data are means ± standard deviations (SDs).

## Results

### Inactivation of MS2 and T4 by plasma-activated mist on face masks

**Figure 2:**
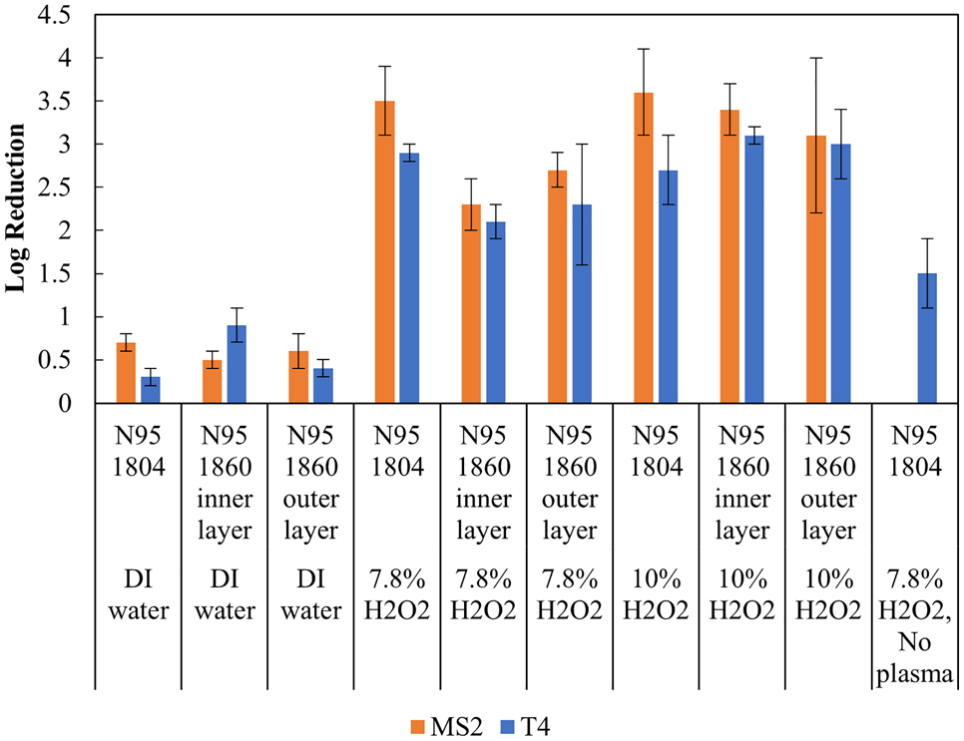
Decontamination efficiency of MS2 and T4 on N95 respirators treated by different compositions of PAM. N95 1804 treated in 7.8% hydrogen peroxide mist without plasma was not tested on MS2. All treatments were found to significantly reduce MS2 or T4 on N95 1804 or 1860 compared to untreated group (*P *< 0.05, independent t-test).

Two types of commonly used N95 respirators were tested in this study. The concentration of MS2 or T4 that inoculated on N95 respirators was in a range from 6- to 8-log PFU/piece. One cycle treatment of N95 respirators coupons in our system with DI water PAM showed 0.5- to 0.7-log reduction of MS2 and no more than 0.9-log reduction of T4 (Fig. 2 and Supplementary material Table S1). When 7.8% and 10% of hydrogen peroxide was used to generate PAM, results showed higher decontamination efficiency of both MS2 and T4. The comparisons of the log reduction indicate significant differences between treatment groups of DI, 7.8% and 10% hydrogen peroxide (ANOVA, *p *< 0.05). N95 respirator treated by 7.8% hydrogen peroxide PAM in our system achieved over 2-log to MS2 and T4. For 10% hydrogen peroxide PAM, our system achieved a 3-log inactivation efficiency which exceeded U.S. Food and Drug Administration (FDA) emergency use authorization (EUA) requests. FDA EUA requested the bioburden reduction validation demonstrating at least 3-log of a non-enveloped virus challenge^23^. One cycle treatment in our system with 7.8% hydrogen peroxide mist but without plasma achieved 1.5-log reduction to T4 on N95 1804, while at same condition but with plasma can achieve 2.9-log reduction. This means plasma can improve the inactivation ability of hydrogen peroxide alone. In addition, there was no significant difference between the log reduction of either bacteriophage on N95 1804 and 1860.

### Chemical Properties of Plasma-activated Mist and Conditions in the Chamber

Commercial test strips were used to qualitatively measure pH, NO_3_^−^, NO_2_^−^ and total peroxide. Even though the results were not quantitatively precise, the use of the commercial test trips provided some insight into the chemistry of the PAM generated (Supplementary Material Figure S3). When DI water was used as the source of PAM, the pH strips indicated the pH of PAM was around 1. While the pH was 0 when 10% hydrogen peroxide was the source of PAM. NO_3 _^−^ and NO_2_^−^ measured in DI water PAM were 500 and 80 mg/L, respectively. Unfortunately, the results of NO_3_^−^ and NO_2_^−^ were inconclusive in 10% hydrogen peroxide PAM because the color of nitrite and nitrate strip appeared bleached after one cycle treatment. In addition to placing these indicator strips in the treatment device, pH, NO_3_^−^ and NO_2_^−^ were also measured using these strips in condensed water collected from PAM generated by DI water (Supplemental material Figure S3), where it was shown that the pH of the condensed water was 1.5, NO_3_^−^ and NO_2_^−^ were > 500 and 1 mg/L, respectively.

**Figure 3:**
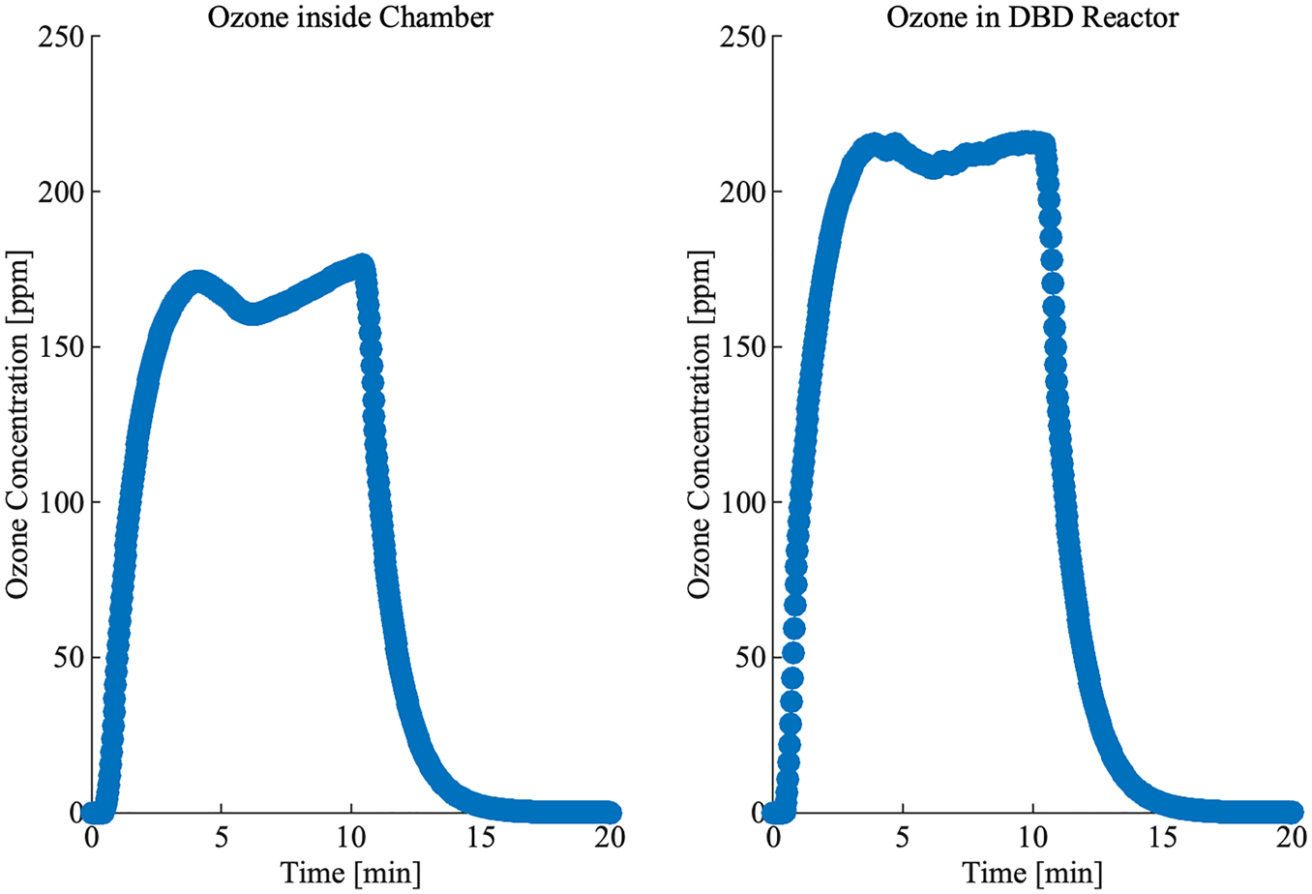
Ozone concentration measured in the sample placed chamber (left) and in the DBD reactor chamber (right) during one operation cycle.

In addition to the chemistry of the droplets, we were also interested in the concentration of reactive oxygen species, such as ozone, within the treatment chamber. Therefore, the concentration of ozone was also measured at two locations in our system during one operation cycle, with one location directly adjacent to the DBD plasma reactor and the second location inside the treatment chamber where masks were placed (Fig. 3). In the chamber, it was shown that the ozone concentration increased drastically and stayed at a high level for the first 10 min. During the next 10 min, the ozone concentration decreased because the DBD plasma reactor was turned off and heating was turned on. The maximum concentration of ozone in the chamber where the masks were placed was around 170 ppm, while the maximum concentration of ozone was 215 ppm in the DBD plasma reactor. During the treatment cycle, the ozone concentration decayed to 0.03 ppm after a completed cycle.

**Figure 4:**
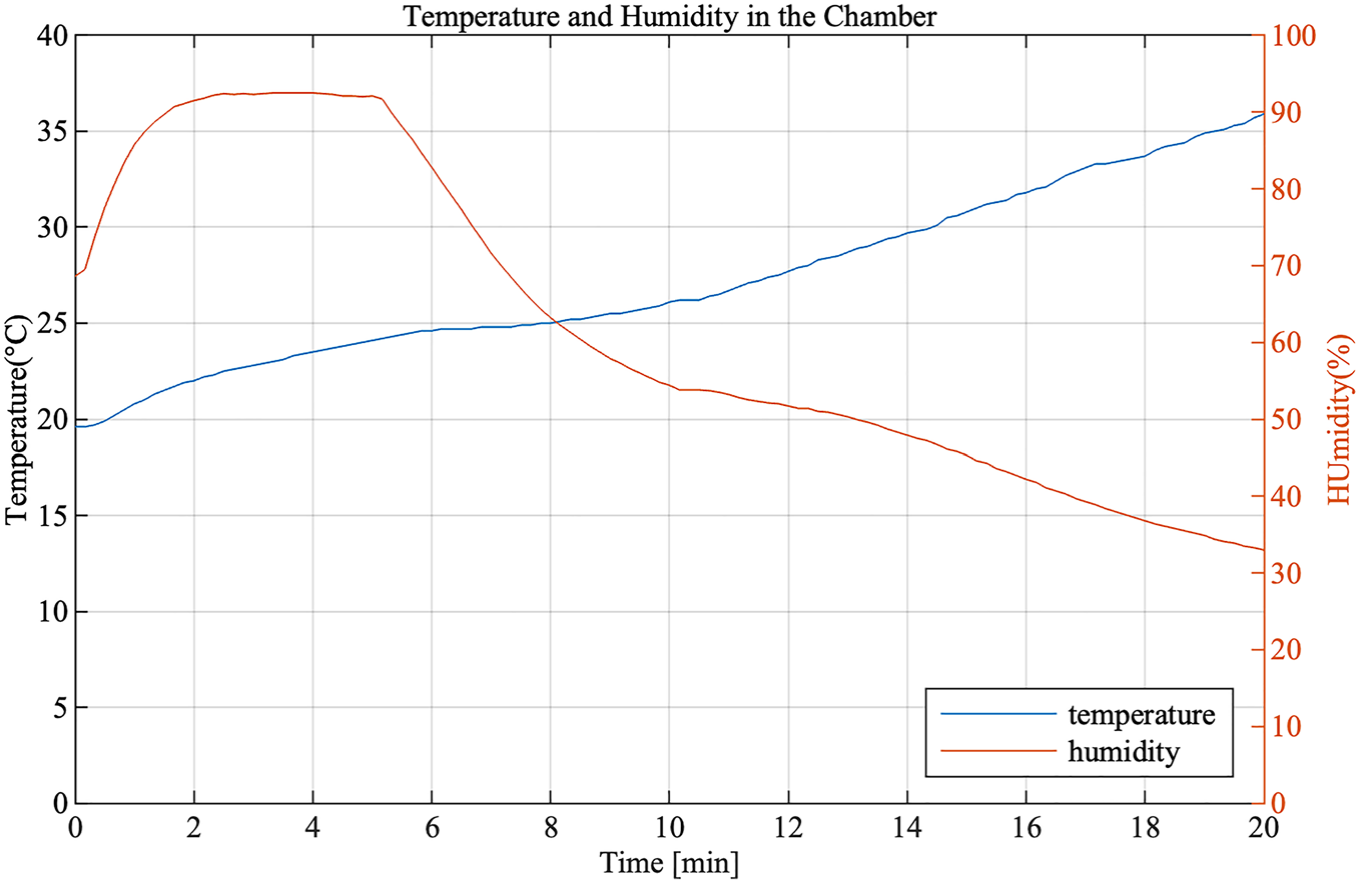
Temperature and humidity of one cycle treatment with DI water PAM.

In addition to ozone, the temperature and humidity in the chamber was also measured (Fig. 4). In Fig. 4, it can be seen during one operation cycle that the temperature steadily increased from below 30 °C to 36 °C, while the humidity increased for the first 5 min up to 90% while the nebulizer operating. When the nebulizer was turned off after 5 min, the humidity decreased down to 33% by the end of the treatment cycle.

### N95 masks quality assessment

The filtration efficiency of two N95 respirators (3 M 1860) were tested before any plasma treatment. Then, one of those masks was treated for 1 cycle in our system with DI water as source of mist, and other one was treated for 20 cycles. Their efficiencies of filtration were tested again. There was no discernable change in mask removal efficiency after 1 or 20 treatments. This result indicates that there is no degradation of the filter material that would affect airborne particle or aerosol removal by the respirator.

Fragmentation of the elastic material in the straps of 3 M 1804 was found after one cycle treatment in our system with DI water alone and with hydrogen peroxide. In contrast, fragmentation was not observed on 3 M 1860 straps even after 20 cycles treatment in 10% hydrogen peroxide. Interestingly, based on the details on their commercial packaging, the straps of 3 M 1860 are made of braided polyisoprene while the straps of 3 M 1804 are made of polyisoprene. Further studies are needed to investigate how PAM, as well as other disinfection technologies, may impact the structural integrity and functionality of respirator straps following repeated sterilization.

## Discussion

The pulsed DBD plasma system used in this study is a small, cost-effective, easy to manufacture and distribute plasma-based disinfection device designed for reusing PPE in a pandemic environment, which can achieve high decontamination efficacy in a short treatment time. Current treatment cycle is fixed at 10 min PAM and 10 min heat drying. It has the potential of reaching higher decontamination levels with using different additives to the liquid and/or changing the plasma parameters (e.g., time of treatment or plasma power). The higher concentration of hydrogen peroxide was shown to increase the inactivation ability, but the quality of N95 masks after treatment will need to be tested to ensure the filtration efficiency is not degraded. Lower concentration of hydrogen peroxide, such as 3%, should be considered in the future as well because of its availability to the general public.

Prior to our study, the National Institute for Occupational Safety and Health (NIOSH) found ultraviolet germicidal irradiation (UVGI) and vaporous hydrogen peroxide (VHP) to be more promising as potential methods to decontaminate N95 respirators^24^. UVGI with different settings have been tested in previous research by treating various microorganisms on N95 respirators, such as *Bacillus subtilis* spores^25^, influenza viruses^8^, SARS-CoV-1 and SARS-CoV-2^26^. UVGI has substantial inactivation ability, but may be discouraged by shadowing effect produced by the multiple layers of the N95 respirators^8^. Meanwhile, UVGI may have a considerable impact on the strap tension and strength of the layers of some N95 mask models^7^. Because our system is a non-thermal disinfection method and the temperature during decontamination cycle is under 36 °C, it can avoid the decrease of filtration performance that caused by heat (at 80 °C)^7^.

The mechanism of how PAM inactivate microorganisms is not clear, but previous research has considered that PAM act as a more concentrated form of PAW which can more efficient transports plasma reactive species to surface because of their high interfacial surface area^16^. In this study, we found high concentrations of nitrate which indicated the formation of peroxynitrite, which has been shown to contribute to microbial inactivation^27^. In addition, we demonstrated in this study that plasma enhances the antimicrobial effectiveness of hydrogen peroxide which agrees with previous research^21,28^. One proposed reason why plasma can enhance the reactivity of hydrogen peroxide is due to the production of hydroxyl radicals (OH*), when hydrogen peroxide combines with ozone in what is referred to as the peroxone process^29^. As shown in Fig. 3, the plasma system is able to produce significant amounts of ozone that can drive the following peroxone reaction:

3 $$H_{2} O_{2} + 2O_{3} \to 2OH^{*} + 3O_{2}$$

In addition to the device used in this study, there exist a number of other ozone-based and hydrogen peroxide-based disinfection devices which have been demonstrated to effectively decontaminate N95 masks, such as STERRAS 100NX (hydrogen peroxide gas plasma), Bioquell Z-2 (VHP), Sterizone VP4 (vaporized hydrogen peroxide-ozone hybrid), and Clēan Flow Mini (UVC, hydrogen peroxide, ozone hybrid)^30^. These devices, except Clēan Flow Mini, need high concentration of hydrogen peroxide solution (30–59%)^31,32^. Although hydrogen peroxide gas plasma, which utilizes 59% hydrogen peroxide in its plasma phase, has been shown to have strong inactivation effect (an average of 5.6-log reduction of MS2), it has also been demonstrated that its use can decrease the face mask filtration efficiency after just 3 cycles^33^. Another the downside of needing high concentrations of hydrogen peroxide is its high costs, as well as policies that highly regulate the transport of such concentrated solutions. Results from this study show that it is possible to design a plasma system with lower hydrogen peroxide concentrations that can achieve sufficient virus inactivation without compromising the filtration efficiency of the N95 mask.

Although the results in this study indicate that PAM can be used to effectively sterilize N95 masks for their potential reuse, there were some limitations in our study. For example, we only tested the inactivation efficiency on filter materials of two types of N95 respirators. Other materials of respirators, such as straps, staples, nose clip, and nose foam, were not tested. Meanwhile, other PPE such as surgical masks, isolation gowns, googles, and face shields, should be tested in the future. These PPE are made of various materials. For example, face shields are manufactured with polycarbonate (PC) and poly(ethylene terephthalate) (PET) sheets^34^. Surgical masks are usually made of same material as N95 masks which is polypropylene (PP)^35^. Disposable isolation gowns are made of synthetic fibers (e.g., polypropylene, polyester, polyethylene), while reusable gowns are typically made of 100% cotton, 100% polyester, or polyester/cotton blends^36^. Two types of non-enveloped bacterial phages MS2 and T4 were tested in this study, but surrogates with more similar structure of SARS-CoV-2 or itself, and gram-positive bacteria with stronger resistance to disinfectants should also be considered in the future. In addition to testing this technology on more viruses or bacteria on various materials, further experiments need to be conducted to determine the chemical properties of PAM. Currently, due to the complexity of plasma and liquid interfacial reactions and the small droplet sizes generated, there is only a qualitative understanding of the chemistry and chemical reaction processes that occur in PAW and PAM from a theoretical modeling perspective^37,38^. More experimental work needs to be done to verify or validate these theories and improve our understanding of the chemistry within PAM and how they can be further utilized for sterilization of materials.

## Conclusions

The sterilization system used in this study is a plasma-based technology that combined surface DBD plasma and water or hydrogen peroxide mist. It can achieve at least a 2-log reduction of MS2 and T4 (two non-enveloped viruses) on N95 respirators treated in one cycle with 7.8% hydrogen peroxide PAM and at least 3-log reduction with 10% hydrogen peroxide. Meanwhile, there was no significant degradation of the filtration efficiency of 3M N95 1860 and 1804 treated after 20 cycles with DI water, 7.8%, and 10% hydrogen peroxide PAM. Although we have evidence that ROS and RNS are present PAM, further studies are needed to confirm the impact of these chemical species on inactivation mechanisms during the use of PAM for disinfection. While there are disinfection technologies that use hydrogen peroxide mist alone, our results indicate that plasma can improve the inactivation efficacy of hydrogen peroxide mist. The short operation time and small size of our device provides a commercial potential to our system.

## Supplementary Information

Supplementary Information.

## Data Availability

All data relevant to the study are included in the article or uploaded as supplementary information.
