# Supplementary material for: Plasma-generated reactive water mist for disinfection of N95 respirators laden with MS2 and T4 bacteriophage viruses
Source: Sci Rep. 2022 Nov 19;12:19944. doi: 10.1038/s41598-022-23660-5 (PMC9675796; doi:10.1038/s41598-022-23660-5)
Supplement: Supplementary file 1 — Supplementary Information. [file 41598_2022_23660_MOESM1_ESM.pdf]

Supplementary Material

Jinjie He*[1], Michael Waring[1], Alexander Fridman[2], Alexander Rabinovich[2], Charles Bailey[3], Gregory Fridman[3], and Christopher M. Sales[1]

1. Civil, Architectural, and Environmental Engineering, Drexel University, Philadelphia, PA
2. C. & J. Nyheim Plasma Institute, Drexel University, Camden, NJ
3. AAPlasma LLC, Philadelphia, PA

Plasma system

Surface dielectric barrier discharge consisted of two 1 mm thick ceramic plates (2" × 4") with silk-screened conductive epoxy on the high voltage side and an aluminum grounded plate on the other side (available from multiple sellers: https://www.amazon.com/Vikye-Generator-Professional-Ozonizer-Poratable/dp/B086YCCJZR). A sinusoidal waveform of 20 kHz, 3 kV was used for the plasma generation.

A 20 mm perforated stainless steel disk, driven by a 113 kHz signal, was used to generate water microdroplets (available from multiple sellers: https://www.amazon.com/gp/product/B073322QF8/ref=ppx_yo_dt_b_search_asin_title?ie=UTF8&psc=1). The water flow rate through this generator was approximately 1 mL/min.

Bacteriophage

SARS-CoV-2 is a lipid membrane enveloped RNA virus and it is extremely harmful and contagious to humans [1]. Due to the high risk of SARS-CoV-2, harmless bacteriophage is recommended use as surrogate. Meanwhile, non-enveloped bacteriophage such as MS2 and T4 is recommended because its high resistance to disinfectants than enveloped virus [2].

Inoculation of MS2 and T4 on N95 respirators

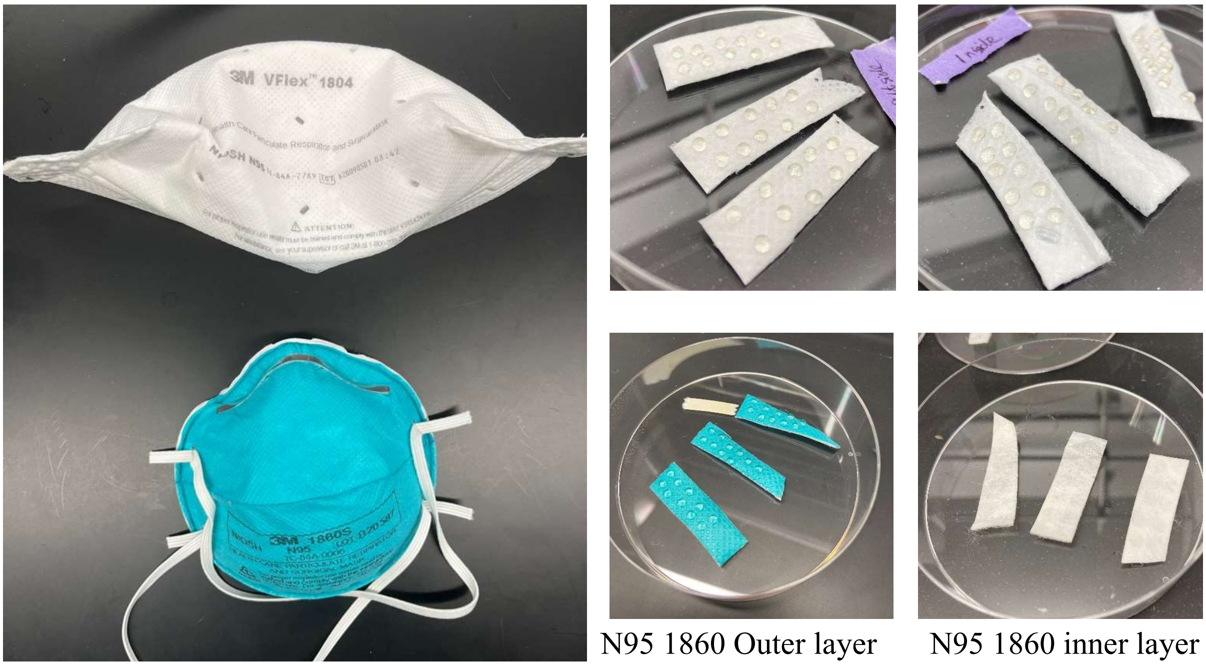

N95 1860 Outer layer N95 1860 inner layer

Figure S1 3M 1804 (upper) and 1860 (lower) whole respirator and cut pieces

Recovery efficiency of MS2 and T4 from N95 respirator material

We compared two virus extraction methods, shaking for 20 min and vertexing for 1 minute in LB broth, on spun bond polyethylene in this study. Spun bond polyethylene is a type of material that commonly used to make the outer layer of surgical masks and N95 respirators. Results showed that when extract T4 right after inoculation from spun bond polyethylene fabric, by shaking coupons in sterile LB broth for 20 min had a higher recovery efficiency (97%, n = 3) than by vertexing for 1 min (89%, n = 2) (p = 0.247, Independent t-test). Also when extracted after 3 hours drying in biosafety hood, the recovery efficiency of shaking for 20 min (7%, n = 4) was higher than vertexing for 1 min (3%, n = 4) (p = 0.297, Independent t-test). So the following experiments were used shaking method because of the higher recovery efficiency.

The recovery efficiency was tested right after and 3 hours after inoculation of T4 and MS2. The recovery efficiency of right after inoculation is greater than 90% for both T4 and MS2 (Table S1). After drying for 3 hours in biosafety hood, the recovery efficiency is around 6%. The lower recovery efficiency was found after 3 hours drying may because viruses penetrated the gaps of mask fabric or died during drying process.

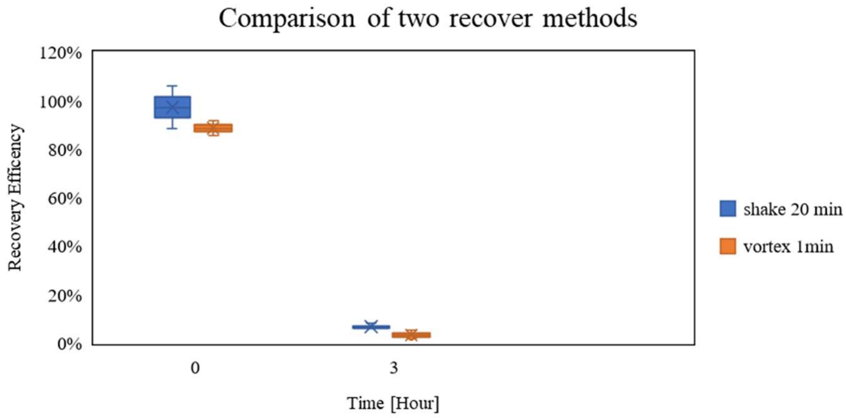

Figure S2 Recovery efficiency of T4 on spun bond polyethylene fabric extracted by shake and vortex in LB broth

Table S1 Recovery efficiency of MS2 and T4 from N95 respirators right after inoculation

Materials	MS2	T4
1860 outer layer	109±12%	91±5%
1860 inner layer	115±14%	100±3%
1804	90±10%	97±13%

Inactivation on N95 respirators after treatment

Table S2 Log reduction of MS2 and T4 on N95 respirators (3M 1804 outer layer, 1860 outer layer and 1860 inner layer) treated by plasma-activated mist generated from DI water, 7.8% or 10% hydrogen peroxide. n is the number of replicates. * indicates that the viability of virus on treated coupons were below the limit of quantification (LOQ) (4500 PFU/coupon for 3M 1804 and 1860 outer layer; 3000 PFU/coupon for 3M 1860 inner layer). The values that below LOQ were replaced by LOQ/2 (2250 PFU/coupon for 3M 1804 and 1860 outer layer; 1500 PFU/coupon for 3M 1860 inner layer) for calculation of log reduction. All treated groups are significant reduced MS2 or T4 on N95 1804 or 1860 compared to untreated group ($P < 0.05$, independent t-test).

	MS2 (mean±S.D.)			T4 (mean±S.D.)		
PAM Source	1804	1860 outer layer	1860 inner layer	1804	1860 outer layer	1860 inner layer
DI	0.7±0.1 (n=6, p<0.001)	0.6 ± 0.2 (n=5, p=0.011)	0.5 ± 0.1 (n=5, p=0.004)	0.3 ± 0.1 (n=5, p<0.001)	0.4 ± 0.1 (n=5, p=0.006)	0.9 ± 0.2 (n=5, p<0.001)
7.8% H_2O_2	3.5±0.4 (n=8, p=0.002)*	2.7±0.2 (n=6, p<0.001)	2.3±0.3 (n=6, p=0.015)*	2.9±0.1 (n=11, p<0.001)*	2.3±0.7 (n=5, p=0.032)	2.1±0.2 (n=5, p<0.001)*
10% H_2O_2	3.6±0.5 (n=13, p=0.005)*	3.1±0.9 (n=9, p=0.008)*	3.4±0.3 (n=6, p<0.001)*	3.2±0.6 (n=9, p=0.023)	3.0±0.4 (n=6, p<0.001)	3.1±0.1 (n=6, p<0.001)
7.8% H_2O_2 mist without plasma				1.5±0.4 (n=6, p<0.001)		

Table S3 Percentage standard deviation of untreated cases

MS2			T4		
1804	1860 outer layer	1860 inner layer	1804	1860 outer layer	1860 inner layer
35%	23%	40%	6%	80%	17%

Quality test of N95 after treatment

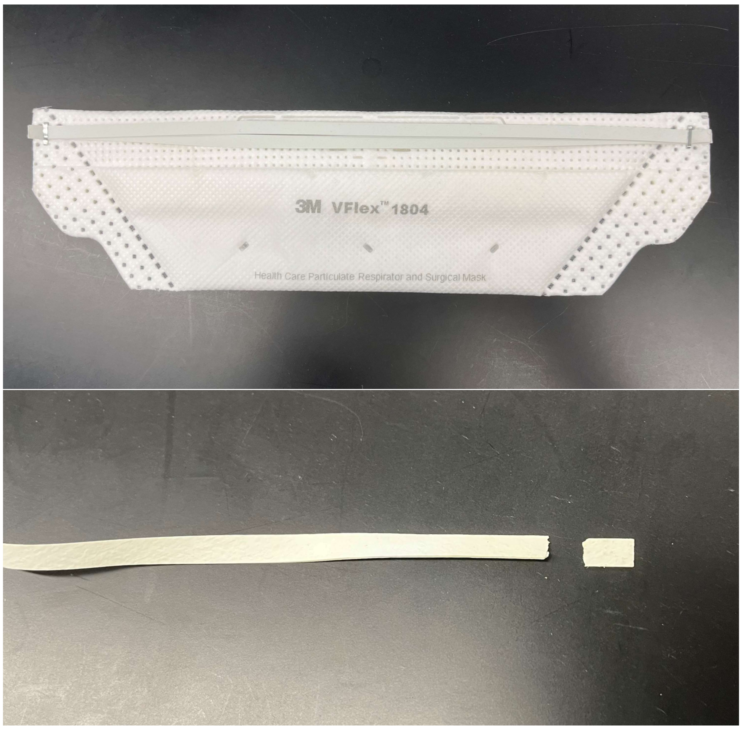

Figure S3 Fragmentation of the elastic material in the straps of 3M 1804. Upper is before treatment, lower is after treatment

Qualitative analysis of chemical compositon in PAM

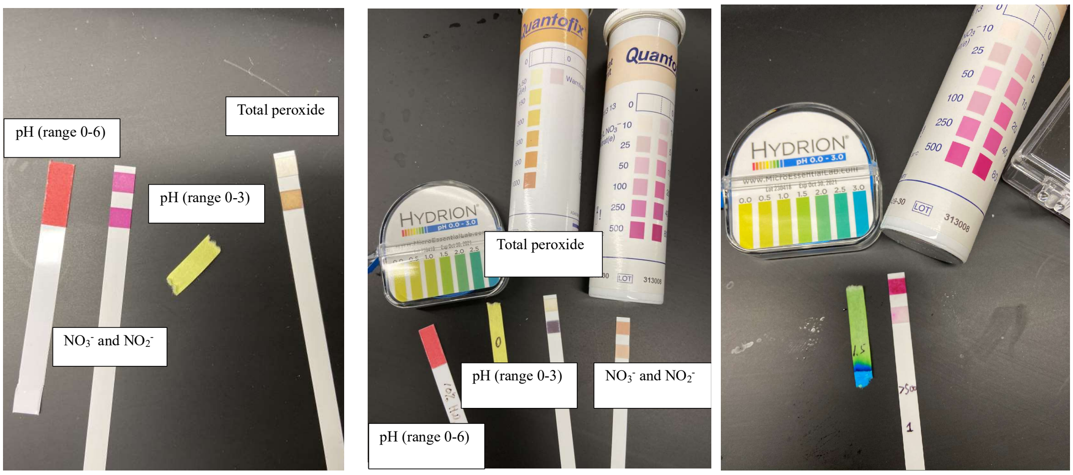

Figure S4 Test strips of one cycle treatment with DI water PAM (left, pH = 1, NO_3^- = 500 mg/L, NO_2^- = 80mg/L, and total peroxide < 50 mg/L) and 10% hydrogen peroxide PAM (middle, pH = 0, total peroxide < 50 mg/L, NO_3^- and NO_2^- were not able to determine). Test strips of one cycle treatment with condensed DI water PAM (right), pH = 1.5, NO_3^- > 500 mg/L, NO_2^- = 1mg/L

References

1. Kumar, S., et al., *Morphology, genome organization, replication, and pathogenesis of severe acute respiratory syndrome coronavirus 2 (SARS-CoV-2).* Coronavirus Disease 2019 (COVID-19), 2020: p. 23.

2. Lucas, W. and D.M. Knipe, *Viral capsids and envelopes: structure and function.* e LS, 2001.
